# Cerebellopontine angle paraganglioma masquerading as vestibular schwannoma

**DOI:** 10.12669/pjms.40.12(PINS).10978

**Published:** 2024-12

**Authors:** Zia ul Rehman Najeeb, Shafqat Ali, Haseeb Mehmood Qadri, Asif Shabbir

**Affiliations:** 1 Dr. Zia Ul Rehman Najeeb, Post Graduate Residents, Department of Neurosurgery, Neurosurgery Unit-I, Punjab Institutes of Neurosciences Lahore, Pakistan; 2 Dr. Shafqat Ali, Post Graduate Residents, Department of Neurosurgery, Neurosurgery Unit-I, Punjab Institutes of Neurosciences Lahore, Pakistan; 3 Dr. Haseeb Mehmood Qadri, Post Graduate Residents, Department of Neurosurgery, Neurosurgery Unit-I, Punjab Institutes of Neurosciences Lahore, Pakistan; 4 Asif Shabbir Associate Professor, Department of Neurosurgery, Neurosurgery Unit-I, Punjab Institutes of Neurosciences Lahore, Pakistan

**Keywords:** Catecholamines, Cerebellopontine angle, Pakistan, Paraganglioma

## Abstract

Paragangliomas are slow-growing, extra-adrenal neuroendocrine tumors with rare intracranial presentation. Although benign, they can be locally aggressive tumors causing bone destruction and compression related symptoms. We report the case of a 19 years old, normotensive female who presented with headache and vertigo for the past six months. Her examination showed right-sided conductive hearing loss and signs of cranial nerve X, vagus nerve palsy. Neuroimaging revealed a lobulated, extra-axial mass measuring 5.4 x 4.2 x 6.8 cm in right cerebellopontine angle (CPA). Subtotal surgical resection was achievable. Histopathology was suggestive of benign, non-secretory paraganglioma. The diagnosis of primary CPA was reached after ruling out other sources of paraganglioma in abdomen, pelvis and thorax. Radiotherapy was advised on follow up visit with no new post-operative deficits seen. Primary CPA paraganglioma should be included in the list of differentials of CPA lesions. Surgical excision in the absence of preoperative embolization of paraganglioma can be successful.

## INTRODUCTION

Paragangliomas are rare, extra-adrenal neuroendocrine tumors. Intra-adrenal paragangliomas constitute a small percentage of adrenal tumors accounting for approximately 1-9% of all adrenal tumors. These are slow growing, benign tumors that develop from embryonic neural crest calls and 95% of them are non-secretory.^1^ Association with hereditary syndromes is seen in >50% of the patients. Hereditary tumors are mostly likely multiple and metastatic; however, sporadic tumors are solitary and unilateral.^1^

Cases of central nervous system paraganglioma are rare and most of them involve cauda equina or filum terminale.^2^ Prevalence of intracranial paraganglioma is low, accounting for only 0.6% of neoplasms of the head and neck region.^3^ In the head and neck, they usually arise in the jugulotympanic region and the middle ear.^2^ There is a slight gender predominance with a female-male ratio of 4: 6.1 for the jugulo-tympanic lesions.^3^ To the best of our compendious search using PubMed and Google Scholar, we report the first case of primary, extra-axial cerebellopontine angle paraganglioma in a young, normotensive female in her teens from Pakistan.

## CASE PRESENTATION

A 19 years old female presented at our outpatient clinic of Department of Neurosurgery in May 2023 with headache for the past six months and associated vertigo and nausea for the three months. Headache was gradual in onset, intermittent, dull in nature and was associated with progressive right-sided hearing loss and difficulty in swallowing. Her past medical and surgical history was unremarkable. She was well-oriented and vitally stable at presentation. Clinical examination showed right-sided conductive hearing loss and absent gag reflex. There were no other sensorimotor deficits. Audiometry confirmed right-sided severe hearing loss of conductive variety. We suspected the presence of intracranial space occupying lesion (SOL), most likely Schwannoma and meningioma.

**Fig.1:**
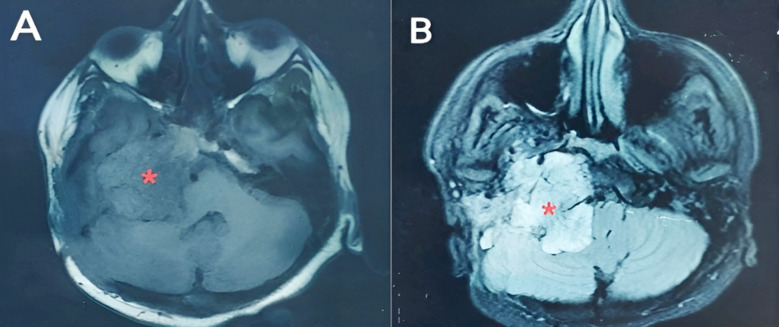
Contrast-enhanced magnetic resonance (CE-MRI) axial slices of brain. A. T1-weighted image and B. T2-weighted image showing hypointense and hyperintense cerebellopontine angle space-occupying lesion, respectively.

She was subjected to a battery of hematological investigations, revealing normal complete blood count, liver function tests, renal function tests and physiological plasma levels of metanephrine and nor-metanephrine. Contrast-enhanced computerized tomography (CE-CT) brain showed a large, ill-defined, enhancing, multi-lobulated, extra-axial SOL measuring approximately 5.4 x 4.2 x 6.8 cm seen in the region of right cerebellopontine angle. Contrast enhanced magnetic resonance imaging (CE-MRI) brain was consistent with the findings of CE-CT; returning signals were hypointense on T1W1, heterogeneous predominantly hyperintense on T2WI and fluid attenuated inversion recovery and was showing homogenous post contrast enhancement with no restricted diffusion on diffuse weighted imaging**/**apparent diffusion coefficient images **(Fig.1)**. Our suspicion was stringly in the favor of Vestibular Schwannoma (VS) after neuromimaging.

Surgical excision of right cerebellopontine angle SOL was achieved through retrosigmoid craniectomy via lazy ‘S’-shaped incision. Intraoperatively, the tumor was yellowish-white, highly vascular and very difficult to suck. Because of highly vascular nature and hard consistency, subtotal resection was achievable. Recovery from general anesthesia was smooth. There were no intra-operative complications. Post-operatively, her gag reflex improved; however, she developed House-Brackman Grade V right-sided lower motor neuron facial palsy and right-sided decreased sensations in the distribution of mandibular division. She was discharged within a week after neuro-monitoring and facial physiotherapy.

**Fig.2:**
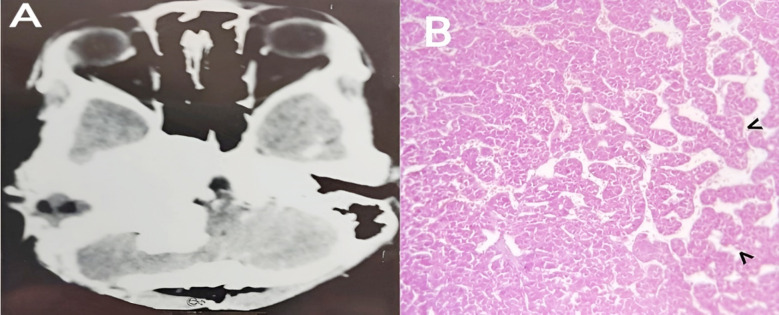
A. Post-operative non-contrast enhanced computerized tomography scan (NCE-CT) brain. B. Hematoxylin and eosin stained histopathology under light microscope - epithelioid cells with abundant, eosinophilic, granular cytoplasm and round to ovoid, salt and pepper nuclei, arranged in a nested (zellballen) pattern shown with black arrow heads and separated by fibrovascular septae with sustentacular cells and rare mitotic activity.

She returned on her follow up visit with improvement in her facial palsy into grade three after physiotherapy. Benign paraganglioma with positivity of smooth muscle actin (SMA) and synaptophysin was the diagnosis upon histopathology and immunohistochemistry (IHC) (Fig.2). NCECT of thorax, abdomen and pelvis were normal and ruled out the presence of paraganglioma in these locations. We concluded it to be a case of primary cerebellopontine angle paraganglioma. Incision wound was healing normally. She was advised to undergo consultation with radiation oncologist at another hospital. She is living a healthy life with improved facial functions until today.

### Consent for publication:

Consent was obtained from the patient for the publication of this case report and the accompanying images.

## DISCUSSION

Development of paragangliomas is attributed to the migration of chromaffin cells into the central nervous system (CNS), where they are not actually found. Failure of foetal migration of neural crest cells could be another probability for their ectopic intracranial presence.^2^ Intercarotidian tissue, jugulo-tympanic region and vagus nerve are the identified sources of origin in head and neck.^2^,^4^

Curry et al. found an equivocal gender distribution with a mean age of 61.2 ± 16.8 years at presentation in their patients with skull base paraganglioma.^5^ However, our patient was a young lady in her teen ages. Paraganglioms have been well studied in association with neurofibromatosis type 1 (NF1), Von Hippel–Lindau (VHL) disease, multiple endocrine neoplasia type 2 (MEN2) and hereditary paraganglioma syndromes.^6^ The young age at presentation could be typically attributed to syndromic presentation of paraganglioma, but our patient had not a combined picture suggestive of any syndrome. Furthermore, due to inadequate funds and lack of appropriate facilities, genetics diagnosis could not be made for our patient. Paragangliomas are typically called “the great masquerades” owing to the diverse facets of their manifestations, ranging from incidental lesions to full blow signs of catecholamine hypersecretion, i.e. hypertension, sweating and tachycardia to mass effects.^7^ As it was an inactive lesion, so our patient did not have the signs of catecholamine toxicity, though mass effects were apparent.

A recently published Pakistani study documents cerebellopontine angle paraganglioma in a 42-year-old female with isointensity on T1, hyperintensity on T2 and a characteristic “salt and pepper” pattern on post contrast enhancement on MRI.^7^ In contrast, our patient has no signs of “salt and pepper” appearance on CE-MRI, which is typical of high vascularity and flow voids of a paraganglioma. We suspected schwannoma in our patient. In such cases, Ota et al. have shown that dynamic susceptibility contrast perfusion (DSC) and diffusion-weighted image (DWI) MRI are significant in differentiating Schwannomas from intracranial paragangliomas. Normalized relative cerebral blood volume and normalized relative cerebral blood flow were significantly higher in paragangliomas than in sporadic/NF2-related schwannomas.^8^

Gross total resection is the gold standard for resect able intracranial lesions without damage to local structure. Most of the time complete resection is not achievable because of high vascularity and haemorrhagic constraints associated with paragangliomas. However, subtotal resection with post-operative radiotherapy is warranted, as postoperative radiotherapy keeps the residual lesion stable and prevents tumour growth.^1^ This is consistent with the management of our patient where gross total resection was not possible and the patient was advised adjuvant radiotherapy.

Preoperative embolization is indicated in surgical excision of head and neck paragangliomas. It is thought to reduce postoperative complications. Gozen et al. conducted a study in Turkey to compare the complications in patients of carotid body paraganglioma with and without embolization. They deduced that embolization did not significantly affect overall complications in both groups, especially the outcomes of vascular injury, cranial nerve injury and hematocrit decrease.^9^ As preoperative embolization was not available at our centre, we also proceeded for surgical excision without embolization and managed the case successfully.

## CONCLUSION

Our case demonstrates the unusual presentation and successful management of cerebellopontine angle paraganglioma in a young female managed surgically without preoperative embolization. Presence of cerebellopontine angle space occupying lesions with extension in jugulo-typmaic region in the absence of characteristic triad of catecholamine hypersecretion and “salt and pepper” pattern on radiology should be suspected as paragangliomas.

### Authors Contribution:

**ZRN** contributed to literature review, data collection, manuscript writing and is responsible for integrity of the study.

**SA** contributed to literature review and manuscript writing.

**HMQ** conceived and designed the study, did literature search, prepared the draft and critically reviewed the article.

**AS** supervised the project and critically reviewed the article.

All authors have approved the final version of the manuscript.

## References

[1] Graham NJ, Smith JD, Else T, Basura GJ (2022). Paragangliomas of the head and neck:a contemporary review. Endocr Oncol.

[2] Xhumari A, Couvelard A, Redondo A, Kalamarides M (2007). Long-term follow-up of an infratentorial primary paraganglioma:a case report. Br J Neurosurg.

[3] Ardeleanu C, Dănăilă L, Arsene D (2005). Paraganglioma of the cerebellopontine angle. Case presentation and pathological considerations. Rom J Morphol Embryol.

[4] Lyne SB, Polster SP, Fidai S, Pytel P, Yamini B (2019). Primary Sellar Paraganglioma:Case Report with Literature Review and Immunohistochemistry Resource. World Neurosurg.

[5] Curry SD, Kocharyan A, Lekovic GP (2023). Multi-Disciplinary Approach to Skull Base Paragangliomas. Brain Sci.

[6] Majewska A, Budny B, Ziemnicka K, Ruchała M, Wierzbicka M (2020). Head and Neck Paragangliomas-A Genetic Overview. Int J Mol Sci.

[7] Akhtar N, Shahid F, Ali AS, Muhammad QUA, Azam NM, Dhakal B (2024). Paraganglioma at the cerebellopontine angle:A case report and review of literature. Clin Case Rep.

[8] Ota Y, Liao E, Capizzano AA, Baba A, Kurokawa R, Kurokawa M (2022). Intracranial paragangliomas versus schwannomas:Role of dynamic susceptibility contrast perfusion and diffusion MRI. J Neuroimaging.

[9] Gözen ED, Tevetoğlu F, Kara S, Kızılkılıç O, Yener HM (2022). Is Preoperative Embolization Necessary for Carotid Paraganglioma Resection:Experience of a Tertiary Center. Ear Nose Throat J.

